# Naltrexone dose-selectively modulates goal-directed behavior and the hypothalamic proteome in rats

**DOI:** 10.1007/s43440-025-00735-4

**Published:** 2025-05-28

**Authors:** Natalia Malikowska-Racia, Przemysław Mielczarek, Piotr Popik

**Affiliations:** 1 Department of Behavioral Neuroscience and Drug Development, Maj Institute of Pharmacology, Polish Academy of Sciences, Smętna 12, Kraków, 31-343 Poland; 2 Laboratory of Proteomics and Mass Spectrometry, Maj Institute of Pharmacology, Polish Academy of Sciences, Smętna 12, Kraków, 31-343 Poland

**Keywords:** Naltrexone, LDN, Motivation, Vigor, Effort-based decision-making, Proteomics

## Abstract

**Background:**

Naltrexone is an opioid receptor antagonist that can modulate reward processing in opposite directions depending on the dose. Whether naltrexone similarly affects motivation remains unexplored. This study investigates the effects of naltrexone on behavioral measures of motivation and search for potential mechanisms, including the endogenous opioid pathway dependent on proopiomelanocortin (POMC).

**Methods:**

Male Sprague Dawley rats received naltrexone (0.01, 0.1, or 1 mg/kg, *ip*) for two weeks. During this period, rats were tested daily using a progressive ratio schedule of reinforcement (PR) test and effort-based choice (EBC) that address motivational vigor, directedness, and effort-based decision-making. After tests, the hypothalami were collected for proteomic analysis using data-independent acquisition (DIA).

**Results:**

Low-dose naltrexone (0.01 mg/kg; LDN) transiently increased PR response vigor without altering decision-making in EBC. At 0.1 mg/kg, but not at the high dose of 1 mg/kg, it impaired effort-based decision-making and goal-directedness. Proteomic analysis correlated LDN with the downregulation of a growth hormone (GH) pathway and altered G protein-coupled receptors (GPCR) signaling. Naltrexone’s intermediate dose predominantly impacted proteins involved in neural growth, while the 1 mg/kg dose affected proteins related to gene regulation.

**Conclusions:**

Different doses of naltrexone had varying effects on motivational measures and the rat’s hypothalamic proteome. Naltrexone 0.1 mg/kg impaired motivational directedness and effort-based decision-making that corresponds to reduced reward signaling due to opioid blockade. In contrast, LDN enhanced vigor, but only early in the treatment. Naltrexone had no effects on the POMC-dependent endogenous opioid pathway, suggesting that a different mechanism underlies its motivational effects.

**Supplementary information:**

The online version contains supplementary material available at 10.1007/s43440-025-00735-4.

## Introduction

Opioid receptors mediate the hedonic impact of rewards in the brain [1]. Blocking these receptors with a relatively high dose of naltrexone, a non-selective antagonist, is commonly associated with reduced reward signaling. This is manifested in rats in decreased sucrose [2] and ethanol consumption [3, 4], reduced drug-induced place preference [5, 6], and cued drug-seeking behavior [7]. Clinical research has shown that naltrexone can reduce neural activity linked to reward-signaling [8, 9], and is particularly effective in individuals who consume ethanol for its rewarding effects [10, 11]. However, naltrexone at lower doses can act oppositely.

Low-dose naltrexone (LDN) paradoxically exaggerates the effects of opioids [12]. It potentiated the rewarding and analgesic effect of opiates [13, 14], prevented morphine-induced tolerance [13] and gliosis [15]. These opposing responses to low- and high-dose naltrexone suggest a more complex mechanism of action than simple antagonism of opioid receptors.

Studies have already suggested that naltrexone can enhance opioid signaling via upregulation of µ-opioid receptors [16, 17], reduced µ-opioid receptors Gs-coupling [18, 19], and increased endogenous opioid signaling [20, 21]. These phenomena prompt new directions for LDN research and can possibly determine its clinical future.

Currently, FDA-approved indications for naltrexone’s treatment solely include alcohol and opioid dependence, as well as auxiliary treatment of obesity [22]. However, LDN has been used as an off-label medication, (for the review see [23]), and the latest studies reported that LDN reduced the severity of post-COVID fatigue syndrome [24, 25] and myalgic encephalomyelitis/chronic fatigue syndrome (ME/CFS) [26].

We hypothesized that naltrexone influences endogenous opioids through the hypothalamic proopiomelanocortin (POMC) pathway [27], and via this mechanism, affects motivation (vigor and directedness) and effort-based decision-making in rats. We examined chronic treatment with naltrexone at the low, intermediate, and standard doses (0.01, 0.1, and 1 mg/kg, (*ip*)) in rats’ operant tests. We used a progressive ratio schedule of reinforcement (PR) that requires a rat to repeatedly press a lever to earn a palatable food reward. The effort-based choice test (EBC) follows a similar design, but the rat chooses between exerting high effort for a high-value reward (palatable food) or opting for less preferred (regular chow) that requires minimal effort.

Motivation is a psychological construct linked to the instigation, maintenance, and persistence of action. At the behavioral level, motivation is understood as a vigorous behavior that is directed toward achieving a specific goal [28–30]. This study used food-motivated operant learning tests in rats to investigate both activational (*vigor*) and directional (*goal-directedness*) aspects of motivation. In our study, the response *vigor* is measured by the number of lever presses in PR. G*oal-directedness* is measured by EBC lever presses as it differentiates reward-directed behavior from primary food drive. *Effort-based choice* is inferred once the number of EBC lever presses is compared with free chow consumption, as it allows to determine whether a rat prefers to work harder for palatable food or chooses less attractive food for free.

To capture the nature of LDN’s effects, we also studied its impact on hypothalamic protein pathways, including endogenous opioid signaling dependent on proopiomelanocortin (POMC) [12]. For this purpose, we performed the proteomic analysis of the rats’ hypothalamus.

## Materials and methods

### Animals

Forty 7-week-old Sprague Dawley male rats (Charles River, Germany) weighing ~ 250 g were used. Animals were group housed (4 per cage) in standard laboratory cages under standard colony A/C controlled conditions: room temperature 21 ± 2 °C, humidity (40–50%), 12-hr light/dark cycle (lights on: 06:00) with *ad libitum* access to water.

Standard laboratory chow (WRF-1, Special Diet Services, UK) was mildly restricted (~ 15 g per animal per day) starting at least one week before the operant training. During operant training, the animals consumed sucrose pellets, and during the tests, they consumed the sucrose pellets (45 mg, Bio-serve, USA) and lab chow. Each day, including the weekends, rats were given ~ 15 g per animal per day of chow starting at 14:00. Thus, the animals were maintained at 85% of their free-feeding weight throughout the study.

Training and testing sessions were done in the morning. All procedures and animals’ maintenance were approved by the II Local Ethics Committee for Animal Experiments at the Maj Institute of Pharmacology, Polish Academy of Science, Kraków, Poland (ethical allowance 3/2022) and followed the European Guidelines for animal welfare (2010/63/EU). This study was planned and performed according to EQIPD guidelines [31]; see https://eqipd-toolbox.paasp.net/wiki/EQIPD_Quality_System and https://quality-preclinical-data.eu/ certificate no. PL-INS-DBNDD-11-2021-1 (Nov 12th, 2021–Nov 30th, 2022); see https://paasp.net/new-eqipd-certified-research-unit/). Animals were randomly allocated to treatment groups; the baseline PR breakpoint was kept similar for all groups.

### Drugs

Freshly prepared naltrexone hydrochloride (Tocris Bioscience, Germany) was dissolved in 0.9% saline (vehicle) and injected *ip* in a volume of 1 ml/kg and administered daily 30 min before tests. The compound was administered at the doses of 0, 0.01, 0.1, or 1 mg/kg for 14 days.

### Operant testing

#### Apparatus

Operant tests were conducted in twelve identical standard operant conditioning chambers (Med Associates, USA), measuring 56 cm × 56 cm × 40.5 cm enclosed in sound- and light-attenuating cubicles. Each operant chamber was connected to a computer through an interface and controlled by MED-PC software (MED Associates). Each chamber was equipped with a food magazine (with photocell beams and light) and one response lever (left or right in odd and even-numbered cages). Food pellets (45 mg, Bio-serve, USA) were delivered via a dispenser connected to the food magazine. A house light was located 17 cm above the top edge of the food magazine. Online control of the apparatus and data collection was performed using MED-PC (Med Associates, USA).

#### Training

**Fig. 1:**
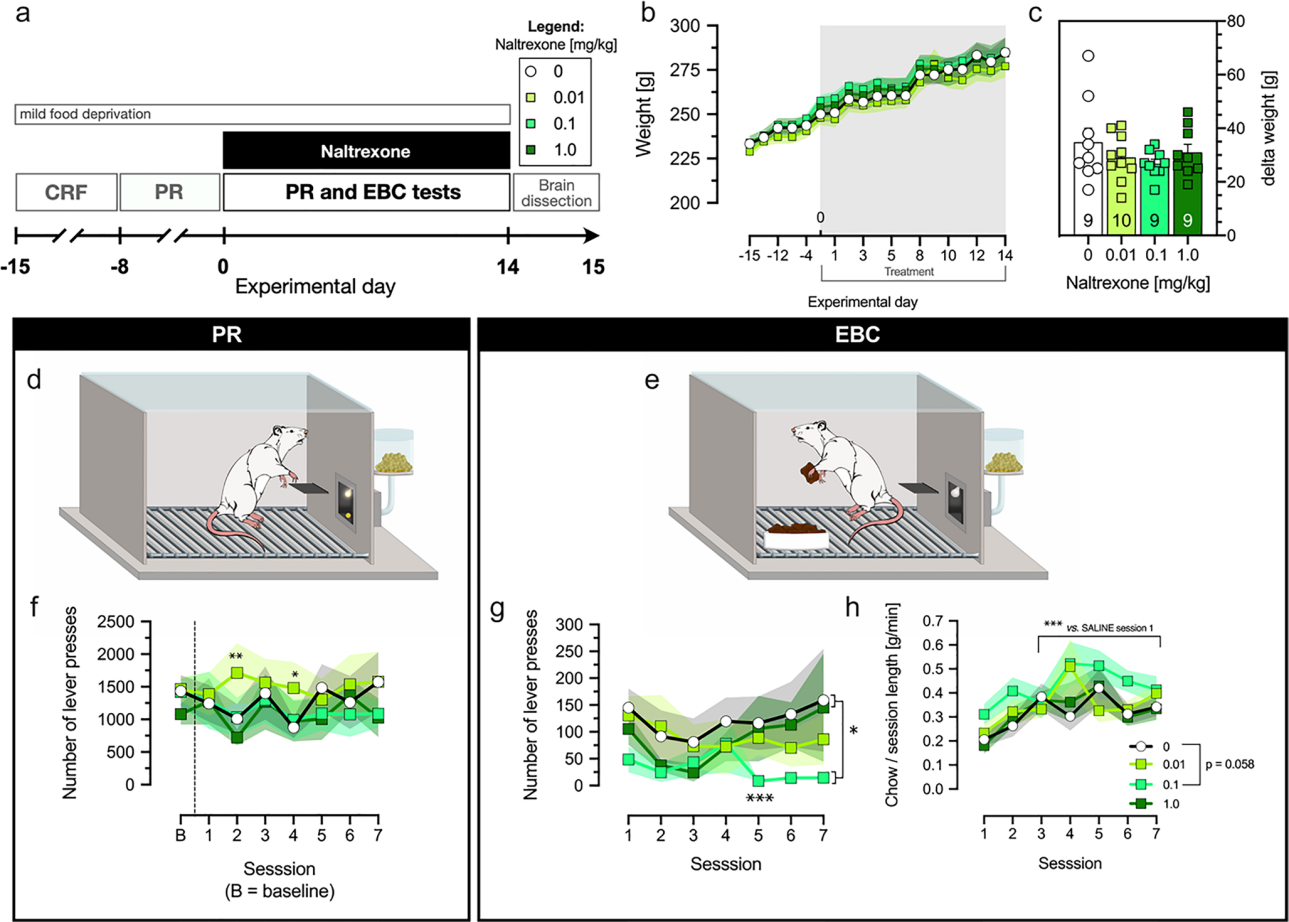
Effect of 2-week treatment with naltrexone on rats’ weight, motivation, and effort-based choice. Experiment timeline (**a**), effects on daily weight (**b**) and gained weight (**c**), schematic representation of rat working in progressive ratio (PR) schedule (**d**) and effort-based choice (EBC) (**e**), the effect of naltrexone on number of active lever presses in PR test (**f**) and EBC (**g**) and the consumption of chow, an alternative low-effort reward in EBC. Data are presented as mean ± SEM. Statistical analysis: repeated measures two-way ANOVA (**b**), one-way ANOVA (**c**) followed by Sidak's post hoc test if applicable (**b, c**), Generalized Estimating Equations (GEE) model followed by Wald chi-square tests (significance of deviations in exponentiated coefficients). Symbols: statistical significance vs. saline * p < 0.05, ** p < 0.01; *** p < 0.001. N indicated within bars in (**c**). B – baseline, CRF – continuous reinforcement schedule, EBC – effort-based choice; PR – progressive ratio

Training began with a single magazine training session (60 sucrose pellets delivered under a fixed ratio 1 and fixed time 60 s schedule). The following day, we introduced the continuous reinforcement schedule (CRF). During a 60-minute CRF session, rats were shaped to press the lever for a pellet delivery. CRF training was continued until all rats met the criterion of 100 pellets/session, and the mean number of active lever presses was > 8 presses/min for at least 75% of rats [32]. Next, PR training sessions were introduced. The number of presses required for a single reward delivery increased by 5 every time 3 rewards at a given ratio were achieved (i.e., 1-1-1 ->5-5-5 ->10-10-10, etc.; based on studies by [33, 34]). PR session lasted for 90 min or was terminated earlier if a rat did not press the lever for 10 min. PR training continued until the breakpoint stabilized, and the mean breakpoint for all rats was higher than 60 (i.e., mean number of lever presses > 1000) for at least 5 consecutive days. Then, the PR and EBC testing, along with naltrexone treatment, started. The scheme of the training progression and the experiment are presented in Fig. 1a

#### Operant tests: progressive ratio (PR) and effort-based choice (EBC)

Rats were subjected to one of the PR (Fig. 1d) or EBC (Fig. 1e) tests per day for 14 consecutive days. Testing sessions were performed alternately, with EBC conducted on odd days and PR on even days. The PR and EBC schedules were adopted from [33, 34] and are based on the lever pressing/chow feeding task originally proposed by John D. Salamone [30, 34, 35]. The PR session was conducted as described in the training. For EBC, the PR operant scheme was employed, but rats were concurrently provided with a ceramic pot filled with the standard laboratory chow (~15 g), placed on the cage’s floor opposite the wall equipped with an operant lever and food magazine. Chow was freely available for rats during the whole EBC session. Once a rat completed the EBC trial, the unconsumed chow was weighed.

#### Proteomic assay

Once behavioral testing was completed, rats were decapitated (~24 h after the last injections), and the hypothalami were dissected. For the analysis, we used four randomly chosen samples from each treatment group. Samples were weighed and then placed in plastic tubes (1 ml) and stored at T = - 80 °C until proteomic assays started.

The protein extracts (15 µg total protein) were reduced in 10 mM tris(2-carboxyethyl)phosphine (TCEP) at 60 °C for 30 min, alkylated by 40 mM iodoacetamide for 30 min at room temperature and finally digested by trypsin (Promega, Rapid-Digestion Trypsin, Mass Spectrometry Grade) applying sp3 method on amine magnetic beads 20 µg/µl (MagReSyn) in KingFisher Flex instrument (Thermo Scientific) at 70 °C for 3 h. Then, samples were acidified by the addition of 10% formic acid and were analyzed on a timsTOF Pro 2 (Bruker) instrument performing data-independent acquisition (DIA) proteomics, operating in positive-ion mode. Peptides obtained by digestion were separated on Ultimate 3000 RSLC nano system (Thermo Scientific) with bioZen C18 nano column 250 × 0.075 mm (Phenomenex) with trap cartridge Acclaim PepMap C18 5 × 0.3 mm (Thermo Scientific) using 60 min. gradient from 2% to 35% of acetonitrile with 0.1% (v/v) formic acid. The mass spectrometer was equipped with an ion mobility separation (IMS) feature, allowing the analysis of ions in a 1/K0 range of 0.6 to 1.6.

Data were acquired over an m/z range of 100 to 1700, and processed for protein identification and quantification. The DIA settings of the mass spectrometer were as follows: 2 × 16 constant isolation windows (2.8 DPPP) with two windows per one parallel accumulation serial fragmentation (PASEF) scan with default settings. The DIA data were processed by DIA-NN version 1.8.1 software [36] using spectra library free search with deep learning and MBR options enabled against a FASTA file containing proteome of *Rattus norvegicus* (rat), including all reviewed (Swiss-Prot) and unreviewed (TrEMBL) proteins (47,921), proteome ID UP000002494. Search parameters were set as follows: modifications: N-term Met excision and Cys carbamidomethylation, up to 1 missed cleavage; peptide charges: from + 1 to + 4; peptide length range: 6–60 amino acids; all other parameters were as default for trapped ion mobility spectrometry (TIMS) with PASEF data.

### Analyses and statistical approach

#### Behavioral studies

To estimate sample size, we employed the G*Power program. Effect sizes were estimated based on our earlier pilot study on amphetamine’s effect in PR and EBC (0.849 and 1.34, respectively). G*Power indicated the required number of at least 8–10 animals/group for PR and EBC, respectively, at p < 0.01. For repeated measures design (and effect size from pilot at 0.7, and 7 repetitions assumed), analyses indicated 8 animals/group (at p < 0.05). Animals were randomly assigned to treatment groups in R.

Iterative Grubbs (Alpha = 0.01) test for outliers indicated three rats based on the number of lever presses under EBC (deviation from mean > 2.5 standard deviations; one from each group: saline and naltrexone 0.1 and 1 mg/kg, details in Supplementary file). The final N after outlier exclusion was 37, with n = 9 for groups treated with vehicle, naltrexone 0.1 and 1 mg/kg and, n = 10 for the group treated with naltrexone 0.01 mg/kg (also see Fig. 1c, numbers within bars indicate N).

PR and EBC lever press data were analyzed using a generalized estimating equations (GEE) approach involving the estimation of marginal models to fit the number of lever presses as a function of treatment and consecutive session number and their interactions [session x treatment] with the assumption of Pascal distribution (discrete and overdispersed data, right-skewed distribution, variance ≫ mean). The statistical significance (p < 0.05) of factors was determined based on estimated model effect, margins, and marginal effects (average outcome variable level in a given group at a given time point) with respect to reference categories (vehicle treatment and first training session) tested with Wald chi-square test. A similar approach was used to test chow consumption [g] during EBC; data were first normalized to session duration (from 10 to 90 min, [g/min]) and then ln transformed for GEE analysis with normal distribution assumed.

GEE model was used instead of classical parametric ANOVA because of the three major reasons listed below (also see supplementary materials). (1) The data (breakpoint, number of lever presses, and pellets gained) were significantly right-skewed, meaning that rats were more likely to perform fewer lever presses, earn fewer pellets, and reach lower breakpoints in both PR and EBC. This violated the assumption of normal data distribution and resulted in high standard deviations, disqualifying simple parametric approaches. (2) Data transformation did not help resolve this issue, and a majority of datasets still deviated significantly from the Gaussian standard. Also, transformations rely on quotients, logarithms, or trigonometric functions, which cannot appropriately handle ‘0’ values. For current experiments, these values convey relevant information about the choices the rats made during tests. (3) Last but not least, the aim here was to retain the repeated measures factor to observe how the effects of naltrexone evolved over time. Instead of performing multiple non-parametric equivalents of one-way ANOVA, we opted for an approach that could assess treatment effects over a longer measurement period, which here manifested in skewed and overdispersed datasets. This type of analysis is facilitated by the GEE model, which allows for the *a priori* definition of data distribution and properly accounts for repeated or correlated data.

Rats’ weight was analyzed using repeated measures ANOVA with Greenhouse-Geisser correction due to violated sphericity assumption, while for delta weight examination, simple one-way ANOVA was employed. If applicable, Sidak’s post hoc test was used for multiple comparison analysis.

For statistical analysis, we used SPSS statistics, IBM SPSS v. 25, and GraphPad Prism v. 8. Specific statistics are provided in the Supplementary Materials. Graphs were created in GraphPad Prism (v. 8), and for artworks, the Affinity Designer (v. 1.10.8) was applied.

#### Proteomic assays

**Fig. 2:**
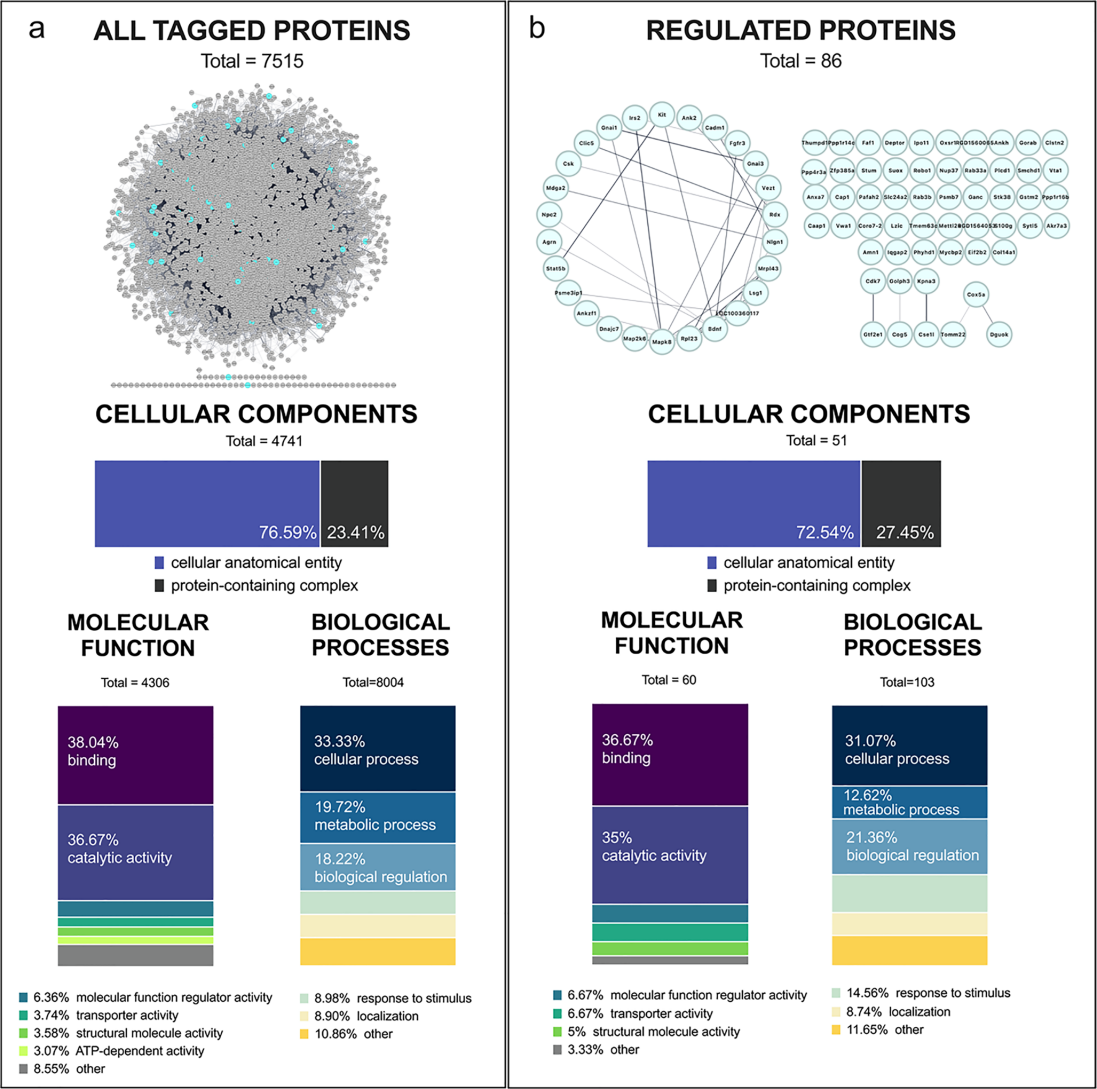
Categorization of all identified hypothalamic proteins (**a**) and proteins that were significantly altered in naltrexone-treated rats (**b**). The top graphs demonstrate the schematic representation of retrieved proteins (Cytoscape STRINGapp). Functional enrichment analysis found no significant differences between regulated proteins and all tagged proteins (background). Bottom stacked bar charts represent the percentage of protein in different categories (PANTHER database, categorization based on Gene Ontology categories (https://geneontology.org/)

Identified proteins were statistically processed in Perseus software version 2.0.10.0 (Max Planck Institute; [37]) was used for data preparation and statistical examination. Data analysis involved log2 transformation and filtering (three out of four valid cases for at least one categorical variable) records. Then, data were subtracted by a median. The effect of naltrexone treatment on peptides’ fold change was examined with one-way ANOVA followed by the Tukey post hoc test. For ANOVA, p < 0.01 was considered statistically significant, and FDR < 0.05 for the post hoc test (Fig. 2, 4 and Table 1). The PANTHER (Protein Analysis THrough Evolutionary Relationships, v. 18; [38]) was used for the categorical (Gene Ontology categories (https://geneontology.org/) adjustment of proteins (Fig. 2). For enrichment analysis and pathway investigation, we used Cytoscape StringApp [39] and searched databases: Brenda Tissue Ontology (BTO),GeneOntology(GO), KEGG (Kyoto Encyclopedia of Genes and Genomes), Reactome, STRING (Search Tool for the Retrieval of Interacting Genes/Proteins), UniProtkeywords, Wikipathways.

Graphs were created in GraphPad Prism (v. 8), and the proteins’ network visualization was done in Cytoscape StringApp based on STRING database.

## Results

### Naltrexone had no effect on the rats’ body weight

Repeated measures ANOVA found that rats’ weight increased by about 20% throughout the experiment (Fig. 1b: F_2.864,94.499_ = 155.655, p < 0.001) but treatment affected neither the weight (F_3,33_ = 0.462, p=ns) nor modified the day’s effect (F_8.591, 94.499_ = 0.485, p=ns). We also examined whether treatment affected the total weight gained throughout the treatment period, and no differences among tested groups were noted (Fig. 1c: F_3,33_ = 0.979, p=ns).

### Effects of naltrexone on motivation and effort-based decision-making

Figure 1d represents a schematic drawing of PR test, and the test results are presented in Fig. 1f. Model (session x treatment) yielded that in PR, the mean number of lever presses differed across sessions (*χ*^*2*^ = 50.295, df = 6, p < 0.0001), and marginal effects indicated a significant reduction on the 4^th^ session (p = 0.001) and marginally significant reduction on 2^nd^ session (p = 0.057), as compared to the first test.

The treatment did not affect lever presses (*χ*^*2*^ = 1.249, df = 3, p = 0.741). However, treatment effect depended on the session (*χ*^*2*^ = 140.644, df = 18, p < 0.0001). On 2^nd^ and 4^th^ sessions, a general reduction was observed, and marginal effects indicated a significant increase in the 0.01 mg/kg naltrexone-treated rats (p = 0.001 and p = 0.01, respectively) as compared to the reference saline.

Figure 1e represents the EBC schematic design, and the test results are shown in Fig. 1g (number of lever presses) and Fig. 1h (chow consumption). Free chow availability led to about a 90% drop in lever press number in EBC as compared to PR (1274.49 ± 114.102 vs. 82.68 ± 42.85, mean ± SEM). The model found significant effects of the session (*χ*^*2*^ = 26.757, df = 6, p < 0.0001), treatment (*χ*^*2*^ = 9.402, df = 3, p = 0.024) and their interaction (*χ*^*2*^ = 112.758, df = 18, p < 0.0001). Marginal effects demonstrated an overall reduction in the number of lever presses for naltrexone 0.1 mg/kg treated rats (p = 0.035) with a significant reduction on the 5^th^ session (p = 0.009) as compared to the saline reference.

Test of model effect demonstrated that normalized chow consumption (Fig. 1h) depended on the session number (*χ*^*2*^ = 44.606, df = 6, p < 0.0001), and that session interacted with the treatment (*χ*^*2*^ = 114.714, df = 18, p < 0.0001). Marginal effects indicated a significant increase in chow consumption after 2^nd^ session and suggested a high but marginally significant increase in naltrexone 0.1 mg/kg treated rats (p = 0.058).

### Proteomic analysis

The raw data matrix included 7632 records; after filtering, 7515 were retrieved, and then log2 was transformed. The statistical analysis revealed that the treatment affected 456 (p < 0.05) and 86 (p < 0.01) proteins. To ensure the highest level of accuracy, we considered only statistically significant effects at p < 0.01 (86 peptides). Between-group effect was examined with Tukey’s post hoc comparisons, for which FDR < 0.05 was considered statistically significant.

### Functional classification and enrichment analysis

The PANTHER qualitative functional classification assigned the 7515 peptides into categories of cellular components (2 categories), molecular function (13 categories), and biological processes (17 categories) listed in the Gene Ontology database. The classification presented in Fig. 2a (only categories containing > 3% of all proteins identified) revealed that proteins were predominantly associated with cellular anatomical entity (76.59%) or protein-containing complex (23.41%). In terms of molecular functions, most peptides were involved in catalytic activity (36.67%), and binding molecular functions (38.04%). Among biological processes, the most frequent were biological regulation (18.22%) or cellular (33.33%) and metabolic processes (19.72%).

The subgroup of proteins (86) that were significantly modulated by naltrexone treatment (one-way ANOVA at cut-off p < 0.01) presented almost identical distribution (Fig. 2b). Peptides were classified into 2 categories of cellular component, 8 categories of molecular functions, and 10 categories of biological processes (127 processes). Cytoscape STRINGapp found no significant functional enrichment in the naltrexone-regulated subgroup of proteins (identified 81 out of 86) as compared to all identified proteins (6868 out of 7515).

**Fig. 3:**
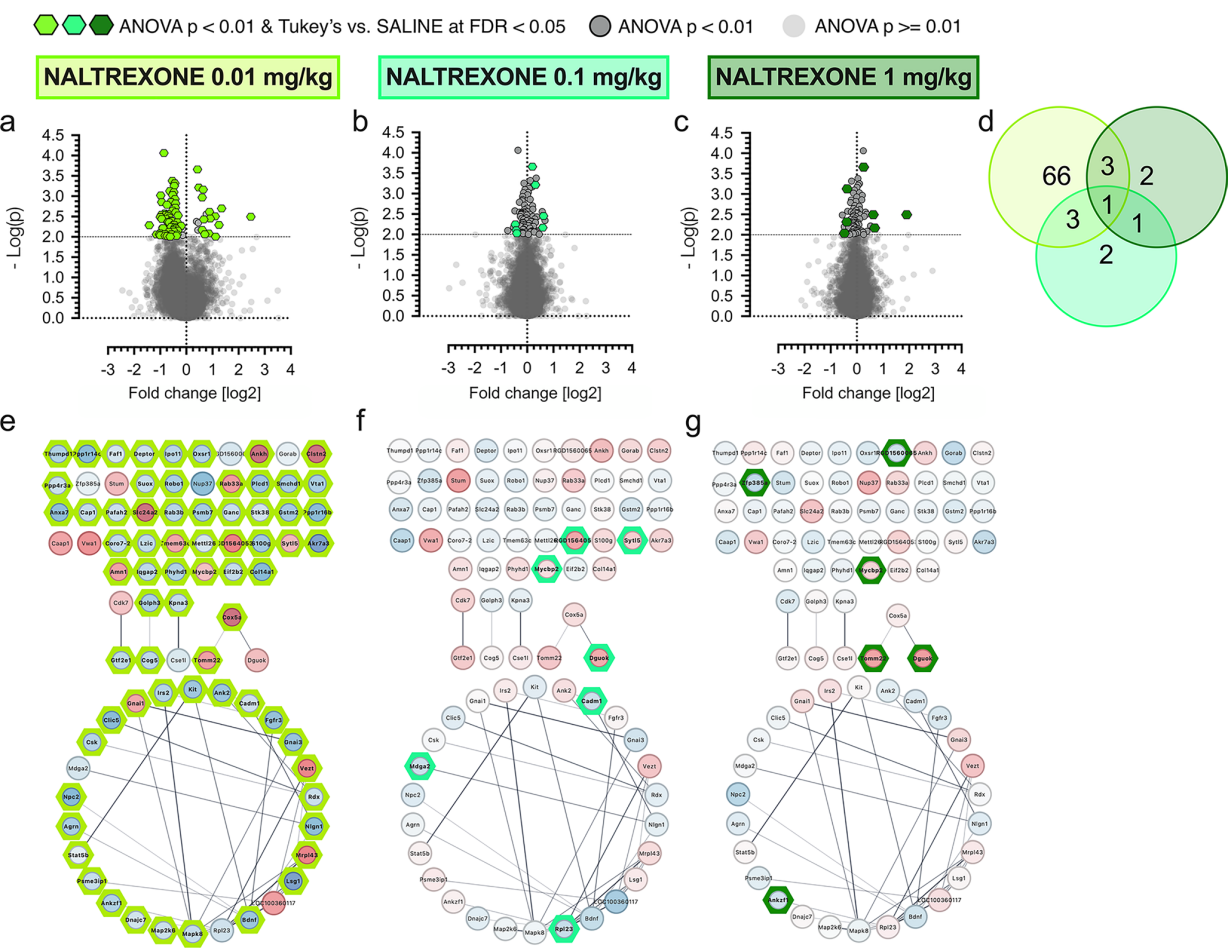
Effects of naltrexone (0.01, 0.1 and 1 mg/kg) on hypothalamic proteins. Starting from left: charts present volcano plots – log(p) vs. log2 fold change as compared to saline-treated group: naltrexone 0.01 (**a**), 0.1 (**b**) and 1 mg/kg (**c**); Venn’s diagram presenting proteins distribution (**d**); schematic representation of specific proteins affected by naltrexone 0.01 mg/kg (**e**), 0.1 mg/kg (**f**) and 1 mg/kg (**g**) with lines indicating protein-protein interaction. Statistical analysis: one-way ANOVA followed by the Tukey post hoc test, cut-off p < 0.01 and FDR < 0.05 considered statistically significant. FDR – false discovery rate

One-way ANOVA (cut-off p < 0.01) demonstrated that treatment with naltrexone 0.01 mg/kg had the most comprehensive effect and regulated levels of 73 peptides, with 58 and 15 peptides down- and up-regulated, respectively, as compared to control (Fig. 3a, FDR < 0.05). Sixty-eight proteins were recognized by the Cytoscape STRINGapp database (Fig. 3e). Analysis indicated that within the group of proteins that differed significantly between control and naltrexone 0.01 mg/kg, the KEGG Pathway of growth hormone (GH) synthesis, secretion and action (rno04935) was enriched (FDR = 0.0251), considering all tagged hypothalamic proteins as a background. Five proteins were downregulated (mitogen-activated protein kinase kinase 6, Q925D6_RAT [*Map2k6*]; signal transducer and activator of transcription 5B, STA5B_RAT [*Stat5b*]; guanine nucleotide-binding protein G(i) subunit alpha-3, GNAI3_RAT [*Gnai3*]; insulin receptor substrate 2, F1MAL5_RAT [*Irs2*]; mitogen-activated protein kinase 8, MK08_RAT [*Mapk8*]), and only guanine nucleotide-binding protein G(i) subunit alpha-1 (GNAI1_RAT [*Gnai1*]) was upregulated as compared to control, suggesting that treatment with naltrexone 0.01 mg/kg led to the pathway inhibition. Naltrexone at doses of 0.1 (Fig. 3bb, f) and 1 mg/kg (Fig. 3cc, g) regulated levels of 7 peptides, with 4 and 3 down- and upregulated, respectively. There was no significant enrichment in any specific pathway for these doses. Figure 3d presents a Venn diagram for proteins significantly altered by naltrexone at different doses.

**Fig. 4:**
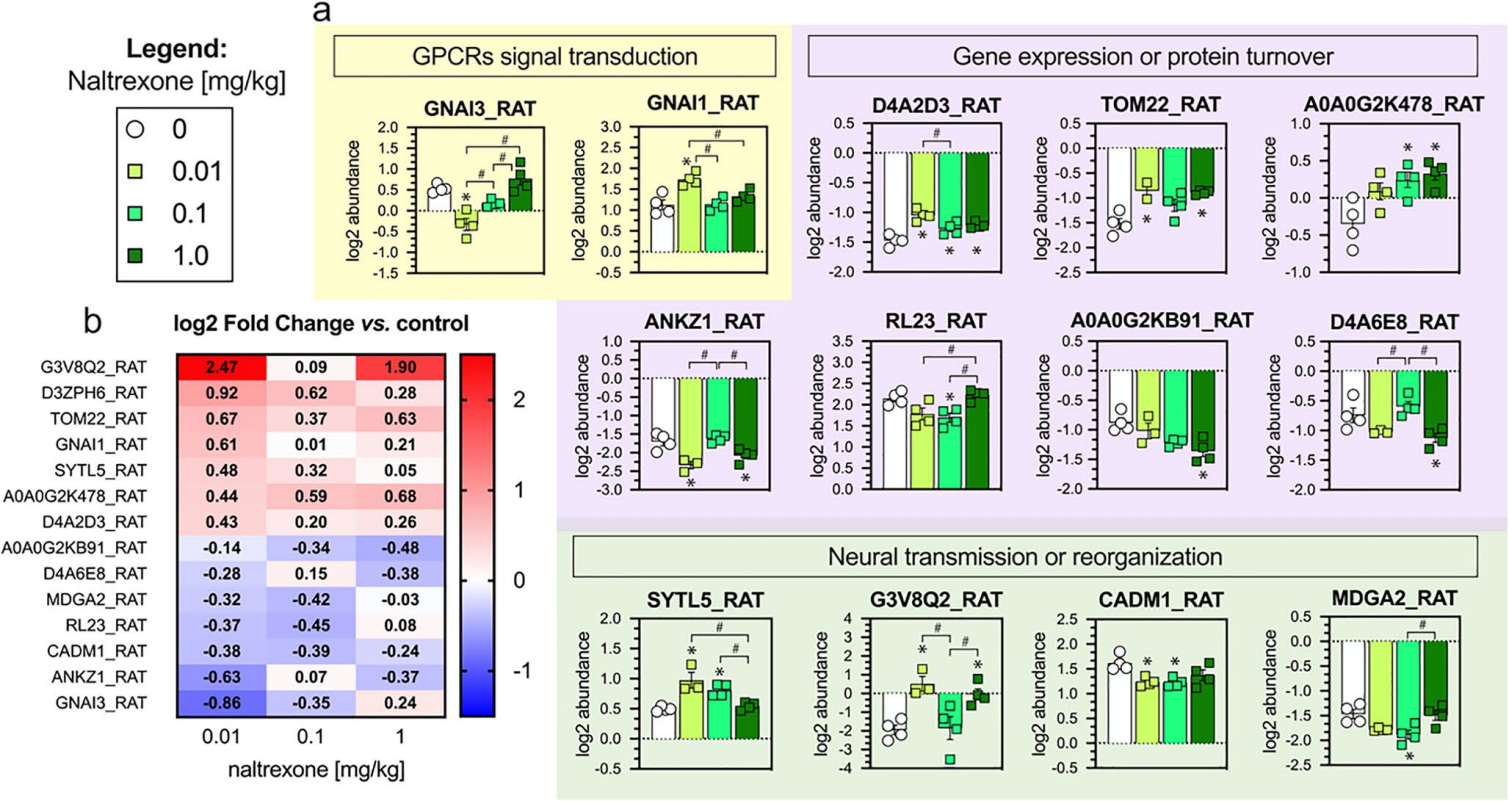
Effects of naltrexone (0.01, 0.1 and, 1 mg/kg) on the levels of selected hypothalamic proteins. Differences presented as absolute abundance value (mean ± SEM) (**a**) and heatmap showing log2 fold change vs. control (**b**). Statistical analysis: one-way ANOVA followed by the Tukey post hoc test, cut-off p < 0.01 and FDR < 0.05 considered statistically significant. Symbols: significance vs. saline * FDR < 0.05; # FDR < 0.05. FDR – false discovery rate, GPCRs – G protein-coupled receptors; UniProt ID: A0A0G2K478_RAT - Deoxyguanosine kinase (*Dguok*); A0A0G2KB91_RAT - Zinc Finger Protein 385A (*Zfp385a*); ANKZ1_RAT - tRNA endonuclease ANKZF1 (*Ankzf1*); CADM1_RAT - Cell adhesion molecule 1 (*Cadm1*); D4A2D3_RAT - RCR-type E3 ubiquitin transferase (*Mycbp2*); D4A6E8_RAT - Similar to human chromosome 1 open reading frame 52 (RGD1560065); G3V8Q2_RAT - Internexin (*Ina*); GNAI1_RAT - Guanine nucleotide-binding protein G(i) subunit alpha-1 (*Gnai1*); GNAI3_RAT - Guanine nucleotide-binding protein G(i) subunit alpha-3 (*Gnai3*); MDGA2_RAT - MAM domain-containing glycosylphosphatidylinositol anchor protein 2 (*Mdga2*); RL23_RAT - Large ribosomal subunit protein uL14 (*Rpl23*); SYTL5_RAT - Synaptotagmin-like protein 5 (*Sytl5*); TOM22_RAT - Mitochondrial import receptor subunit TOM22 homolog (*Tomm22*)

Proteins regulated by naltrexone 0.1 and 1 mg/kg, and proteins which level significantly differed in at least three out of four tested groups (Tukey’s post hoc test, FDR < 0.05) were selected for detailed analysis. We distinguished 13 proteins (Fig. 4 and Table 1). For the sake of clarity, we distinguished three customized categories based on their function listed in the UniProt database ([40]; https://www.uniprot.org): (1) *“gene expression or proteins turnover”,* (2) “*neural transmission or reorganization*,” and (3) “*G protein-coupled receptors [GPCRs] signal transduction*”. Each protein was assigned to a single category (Table 1).

Figure 4a demonstrates the particular abundance (ANOVA at p < 0.01 and Tukey post hoc vs. control at FDR < 0.05), and Fig. 4b presents the same results as the fold change versus the control group (heat map).

We observed that naltrexone in each tested dose increased RCR-type E3 ubiquitin transferase (D4A2D3_RAT [*Mycbp2*]). Naltrexone, inversely proportional to the dose, reduced the level of GNAI3_RAT [*Gnai3*], but only for the dose 0.01 mg/kg, this effect was significant as compared to the control. At the lowest dose, 0.01 mg/kg, naltrexone also upregulated GNAI1_RAT [*Gnai1*], as compared to all other tested groups.

The unique effects of naltrexone 0.1 mg/kg were downregulation of large ribosomal subunit protein uL14 (RL23_RAT [*Rpl23*]) and MAM domain-containing glycosylphosphatidylinositol anchor protein 2 (MDGA2_RAT [*Mdga2*]).

The common effect of naltrexone 0.1 mg/kg and 0.01 mg/kg were increased levels of synaptotagmin-like protein 5 (SYTL5_RAT [*Sytl5*]) and RIKEN cDNA A830018L16 gene protein (D3ZPH6_RAT [*C5h8orf34*]), and reduced levels of cell adhesion molecule 1 protein (CADM1_RAT [*Cadm1*]).

Naltrexone at the highest tested dose, 1 mg/kg, selectively decreased the level of similar to human chromosome 1 open reading frame 52 protein (D4A6E8_RAT [*C2h1orf52*]) and zinc finger protein 385A (A0A0G2KB91_RAT [*Zfp385a*])

The common effect of naltrexone 1 mg/kg and 0.1 mg/kg was upregulated deoxyguanosine kinase (A0A0G2K478_RAT [*Dguok*]).

The shared effect of the 0.01 and the 1 mg/kg dose of naltrexone were upregulated internexin neuronal intermediate filament protein alpha (G3V8Q2_RAT [*Ina*]) and mitochondrial import receptor subunit TOM22 homolog (TOM22_RAT [*Tomm22*]), and reduced level of tRNA endonuclease ANKZF1 (ANKZ1_RAT [*Ankzf1*]), which filters incompletely synthesized polypeptides for degradation.

**Table 1:** Summary of selected hypothalamic proteins regulated by 2-week naltrexone treatment. Table includes summary on proteins presented in Fig. 4 (proteins modified by naltrexone at doses of 0.1 mg/kg and 1 mg/kg and proteins with significantly different levels in at least three out of four tested groups). Statistical analysis: one-way ANOVA followed by the Tukey post hoc test, cut-off p < 0.01 and FDR < 0.05 considered statistically significant. GPCRs – G protein-coupled receptors, N/A – not available

UniProtKB accession number	Protein	Gene	Category	General function	The dose of naltrexone [mg/kg]			UniProt entry
					0.01 (LDN)	0.1	1	
D4A2D3_RAT	RCR-type E3 ubiquitin transferase	*Mycbp2*	Gene expression or protein turnover	protein modification and ubiquitination	↑	↑	↑	D4A2D3
TOM22_RAT	Mitochondrial import receptor subunit TOM22 homolog	*Tomm22*	Gene expression or protein turnover	regulation and translocation of preproteins into the mitochondria	↑		↑	Q75Q41
ANKZ1_RAT	tRNA endonuclease ANKZF1	*Ankzf1*	Gene expression or protein turnover	endonuclease in RQC pathway releases incomplete polypeptides for degradation	↓		↓	Q66H85
A0A0G2K478_RAT	Deoxyguanosine kinase	*Dguok*	Gene expression or protein turnover	regulates gene expression and purine metabolism		↑	↑	A0A0G2K478
RL23_RAT	Large ribosomal subunit protein uL14	*Rpl23*	Gene expression or protein turnover	large ribosomal subunit component responsible for the protein synthesis		↓		P6283
A0A0G2KB91_RATB2RYC6_RAT	Zinc Finger Protein 385A	*Zfp385a*	Gene expression or protein turnover	regulates gene expression			↓	B2RYC6
D4A6E8_RAT	Similar to human chromosome 1 open reading frame 52	*RGD1560065*	Gene expression or protein turnover	purportedly related to gene expression			↓	D4A6E8
SYTL5_RAT	Synaptotagmin-like protein 5	*Sytl5*	Neural transmission or reorganization	vesicle trafficking and neurotransmitter release	↑	↑		Q812E4
G3V8Q2_RAT	internexin	*Ina*	Neural transmission or reorganization	cell differentiation and synaptic transmission	↑		↑	P23565
CADM1_RAT	Cell adhesion molecule 1	*Cadm1*	Neural transmission or reorganization	cell-cell adhesion, neuronal migration and axon growth	↓	↓		Q6AYP5
MDGA2_RAT	MAM domain-containing glycosylphosphatidylinositol anchor protein 2	*Mdga2*	Neural transmission or reorganization	cell-cell interactions, synapse development		↓		P60756
GNAI1_RAT	Guanine nucleotide-binding protein G(i) subunit alpha-1	*Gnai1*	GPCRs signal transduction	G-protein dependent signal transduction	↑			P10824
GNAI3_RAT	Guanine nucleotide-binding protein G(i) subunit alpha-3	*Gnai3*	GPCRs signal transduction	G-protein dependent signal transduction	↓			P08753
D3ZPH6_RAT	N/A	*RGD1564053*	Hypothetical protein	N/A	↑	↑		D3ZPH6

## Discussion

We showed that the effects of naltrexone on motivation (vigor and goal-directness) and effort-based choice in rats depend on the dose. LDN (0.01 mg/kg) temporarily boosted rats’ vigor in the progressive ratio (PR) task at the beginning of treatment, while downregulating the GH protein pathway and altering GPCR-related signaling after two weeks of administration. At an intermediate dose (0.1 mg/kg), naltrexone impaired directional aspect of motivation and tended to shift the rats’ effort-based choice toward less palatable but easier-to-access food. It reflects the typical effect expected of opioid antagonists in reducing reward-driven behaviors. These impairments were temporally correlated with altered levels of proteins associated with neural growth and reorganization in the hypothalamus. Interestingly, the corresponding effects were absent with naltrexone at the highest dose tested (1 mg/kg), which, after two weeks of treatment, primarily affected proteins related to gene regulation.

### The effect of naltrexone on food-motivated behavior depends on the dose

Present data are consistent with previous research demonstrating that naltrexone at 0.1 and 1 mg/kg (*ip*) did not alter total consumption or preference for sucrose vs. chow in SD rats [41].

However, we report that the low dose (LDN, 0.01 mg/kg) of naltrexone transiently enhanced activational (*vigor*) but not directional (*goal-directedness*) aspect of motivation. LDN neither altered effort-based decision-making. LDN effect on vigor was not observed following the initial dose, purportedly consistent with [42] Carlson et al. (2021), who reported a similar failure of a single low naltrexone dose following microinjection into the nucleus accumbens (NAc) in the EBC.

To reiterate, this study reports modest motivational effects of LDN restricted to the PR test, suggesting that the experimental conditions were demanding. Nonetheless, our finding corresponds with human studies suggesting the motivation-enhancing potential of LDN, which augmented the effects of dopaminergic antidepressants and reduced depression scores in the clinic [43].

In contrast, naltrexone at a dose 50 times higher has been found to have no effect on depression scores, caused fatigue [44, 45], impaired effort-based choice [45], decreased hedonic capacity for food reward [46] and worsened emotional blunting [47–49].

These human finings correlate with our observation on regular-dose naltrexone that impaired motivational goal directness and effort-based decision-making. Similar rodent research and LDN studies are still limited. Previous literature (see Introduction) demonstrated that regular dosing naltrexone limits the reward signaling, while the LDN acts in opposite way and potentiate the rewarding effect of opioids. These considered with our results suggest that the effects of naltrexone on motivation and effort-based decision making may be driven via altered reinforcing capacity of the reward.

Thus, our findings need further research, yet thoroughly tested offers a new treatment paths for targeting e.g., apathy, which is a common comorbidity of many psychiatric conditions [50].

### Naltrexone affects the growth hormone hypothalamic pathway but not POMC-dependent endogenous opioid signaling

In this study, we examined naltrexone’s motivational effects in the context of hypothalamic endogenous opioids, aligning with the concept introduced by Brown and Panksepp (2009). Specifically, we assumed that naltrexone can influence endogenous opioids through the hypothalamic proopiomelanocortin (POMC) pathway [27]. POMC-positive neurons in the arcuate nucleus of the hypothalamus release beta-endorphin, which promotes the rewarding effect of food. Another product of POMC cleavage is alpha-melanocyte-stimulating hormone (alpha-MSH), which mediates the POMC anorectic effect. Importantly, the synthesis of alpha-MSH is regulated by autoinhibitory feedback from beta-endorphin [27]. This feedback limits the temporary anorectic effect of naltrexone [51].

Yet, our study suggests that the motivational effects of naltrexone do not depend on POMC-mediated opioid signaling. However, this does not rule out the possibility that endogenous opioids contribute to naltrexone’s motivational effects, potentially through altered downstream opioid signaling within other brain regions.

Naltrexone, by blocking µ-opioid receptors, can attenuate the firing of dopaminergic neurons in the ventral tegmental area (VTA) [52, 53], thereby indirectly influencing the release of mesolimbic dopamine, a key driver of motivation [54, 55]. It aligns with our observation on impaired EBC following treatment with naltrexone intermediate dose. Whether chronic LDN induces neuroadaptation within µ-opioid receptors, as previously suggested [18–21], and thus subsequently alters the opioid–dopamine loop, remains unexplored. Nonetheless, such research would provide a foundation for investigating the purported paradoxical effects of LDN and could help explain our observation of transiently increased vigor. For now, this remains a significant research gap that warrants thorough investigation in future studies.

However, LDN produced the most significant effect on the hypothalamic proteome, while two higher doses had a modest impact. We found that LDN, but not the higher doses, enriched and downregulated the hypothalamic GH pathway, which is known to increase food intake [56]. This observation aligns with previous findings that opioids enhance the GH effects, while opioid receptor antagonists inhibit them [57]. Present observations reinforce the statement that the effects of naltrexone on food consumption depend on the dose and cannot be solely attributed to GH pathway activity. Considering the earlier discussion of the transient POMC-dependent anorectic effect, this suggests that the general decrease in food consumption cannot explain naltrexone effects in operant tests.

### Naltrexone dose-selectively regulates specific proteins

To further investigate the effect of naltrexone on the hypothalamic proteome, we performed a detailed analysis of the selected proteins (Table 1). The analysis of specific proteins revealed that naltrexone significantly affects the levels of proteins related to *“gene expression or proteins turnover”,* “*neural transmission or reorganization*,” and “*GPCRs signal transduction*”.

This analysis suggested that LDN altered proteins involved in *GPCR signal transduction* and *neural growth and reorganization*. Naltrexone at 0.1 mg/kg predominantly affected proteins associated with *neural growth and reorganization*, whereas the dose of 1 mg/kg significantly impacted proteins involved in *the regulation of gene expression*.

#### General effects of naltrexone

Specifically, naltrexone, regardless of the dose, increased the levels of D4A2D3_RAT, a protein that regulates post-translational protein modification and ubiquitination. Naltrexone also exhibited an inverse dose-dependent reduction of GNAI3_RAT, which is a down-stream transducer of Gi protein-coupled receptors signaling cascades and plays a role in opioid receptors and GH signaling [58].

#### GPCRs signal transduction

LDN altered guanine nucleotide-binding protein G(i) subunits by decreasing the level of GNAI3_RAT while increasing the level of GNAI1_RAT. This indicates that LDN uniquely affects the G(i) protein [59] that transmits µ-opioid receptor signaling [58]. This phenomenon may mediate some effects of LDN since different doses neither impact GNAI3_RAT and GNAI1_RAT nor improve rats’ motivation.

#### Neural growth and reorganization

LDN did not affect any protein exclusively, but showed some overlap with naltrexone’s effects at doses ten and a hundred times higher. Both LDN and naltrexone at 0.1 mg/kg decreased the level of 1 CADM1_RAT, a protein involved in Ca^2+^-independent cell adhesion, neuronal migration, and axon growth. At these doses, naltrexone also increased the level of SYTL5_RAT, linked to nucleocytoplasmic and vesicle-mediated transport, and D3ZPH6_RAT, an uncharacterized protein. Naltrexone at the lowest and the highest dose tested increased the level of G3V8Q2_RAT, which is involved in neuronal growth. The 0.1 mg/kg dose of naltrexone uniquely downregulated MDGA2_RAT that is critical for cell adhesion, neurodevelopment, and synapse formation.

In summary, LDN increased the levels of 2 proteins and decreased the level of 1 protein involved in neural growth and reorganization. At the highest dose of 1 mg/kg, naltrexone upregulated 1 protein without any downregulation. Naltrexone at 0.1 mg/kg increased the level of 1 protein but decreased the levels of 2, including a unique downregulation of MDGA2_RAT, which plays a role in neural development and synapse formation.

This pattern suggests that the LDN and the highest dose of naltrexone likely promote neural growth and reorganization, while the middle dose impairs these processes. This observation aligns with behavioral observation that only naltrexone 0.1 mg/kg impairs effort-based choice.

#### Regulation of gene expression and protein turnover

Naltrexone at all tested doses reduced the level of D4A2D3_RAT, as described in the section *general effect of naltrexone*.

LDN had no unique effect on proteins regulating gene expression. However, naltrexone administered at dose a 100 times higher (1 mg/kg) demonstrated a significant impact on proteins involved in gene expression and metabolism, upregulating TOM22_RAT and downregulating ANKZ1_RAT.

There were no common effects between LDN and naltrexone 0.1 mg/kg on proteins regulating gene expression and protein turnover, except for D4A2D3_RAT. The common effect between naltrexone at 0.1 mg/kg and 1 mg/kg was an increase in A0A0G2K478_RAT, a protein involved in gene expression and purine metabolism. At 1 mg/kg, naltrexone uniquely downregulated D4A6E8_RAT and A0A0G2KB91_RAT, while at 0.1 mg/kg, it caused a unique downregulation of uL14RL23_RAT, a protein involved in ribosomal protein synthesis.

Briefly, LDN and naltrexone 0.1 mg/kg upregulated 2 proteins and downregulated 1 protein involved in regulating gene expression and protein turnover. At the highest dose of 1 mg/kg, naltrexone upregulated 3 proteins and downregulated 3 proteins.

This suggests that naltrexone’s impact on protein synthesis may increase with dose, with higher doses affecting more proteins involved in gene expression and protein turnover.

## Conclusions

Our findings indicate that the dose of naltrexone determines its motivational effects in rats. We report that only LDN (0.01 mg/kg) leads to a modest increase in motivational vigor while the tenfold increase in dose (0.1 mg/kg) had no effects on vigor but further impaired directedness of motivation and effort-based decision-making. This study found no correlation between naltrexone’s motivational effects and POMC-depended synthesis of endogenous opioids. It links LDN, but not specifically its motivational efficacy, with G(i) protein signaling and growth hormone, whereas motivational deficits associated with higher dose correlate with impaired neural growth and reorganization. Our study suggests that a different primary mechanism likely drives the motivational efficacy of naltrexone. An alternative, unexplored hypothesis is the involvement of altered downstream µ-opioid receptor signaling, potentially targeting mesolimbic dopamine. Although our results encourage further research on LDN, they do not provide a solid support for the previously proposed hypothesis of LDN-induced enhancement of reward signaling and motivation [13, 14].

### Limitations

Operant effort-based choice tasks in rats in the original [34] and modified form [33] investigate a *resultant* of the effort and reward value on decision-making, as different types of effort (lever pressing or no effort) lead to different rewards (sucrose pellet or chow). This design effectively captures the role of dopaminergic neurotransmission in vigor and decision-making [30], but its suitability for evaluating other drug classes is less explored. The typical “lever pressing for sucrose pellet” versus “no effort for chow” setup under a PR schedule may be insufficient to detect the effects of naltrexone. For instance, the operant schedule used in EBC can influence the impact of dopamine-depleting tetrabenazine, which impairs effort-based choice under an FR5 but not a PR schedule [60, 61]. In our study, we used the PR variant [32, 33], which avoids prior pharmacological impairment and better detects high-effort–promoting drugs.

Operant tasks used here depend on food consumption, so decreased appetite can bias their results [62]. Naltrexone presents only a temporary anorectic effect due to ghrelin and GH modification [63, 64] and negative feedback on alpha-MSH [51]. It should be noted that while naltrexone alone may have limited anorectic effects, the addition of bupropion enhances POMC neuron firing, overcomes restricted β-endorphin release [51, 65], and enables a sustained anorectic response [66].

Here, we examined both the acute and chronic effects of naltrexone; however, the proteomic analysis included samples only from animals that received chronic treatment. This approach was based on the expectation that adaptive changes, rather than the acute effects of LDN, would influence endogenous opioid signaling and contribute to increased motivation. Nevertheless, we observed only a transient effect of LDN at the beginning of the treatment, while the proteomic samples were collected after two weeks of treatment. This suggests that a distinct yet transient mechanism may underlie LDN’s efficacy, which was not captured in this study.

In this study, we investigated a specific hypothesis focused on proteomic changes within the hypothalamus. We observed that naltrexone alters the hypothalamic proteome, but its behavioral effects in PR and EBC show only a moderate correlation with these changes, suggesting the involvement of another primary mechanism. Although a sample size of N = 4/group is commonly used in proteomic analyses, it reduces power to detect more subtle effects. Future studies should therefore consider larger sample sizes and include further validation using targeted approaches such as western blotting.

## Electronic supplementary material

Below is the link to the electronic supplementary material.

Supplementary Material 1

## Data Availability

Raw data and the results of analyses are available in the public repository Mendeley Data (https://data.mendeley.com/) under DOI: 10.17632/dxrfgrz9hf.1.

## Abbreviations

alpha-MSH: Alpha-melanocyte-stimulating hormone
Ankzf1: Ankyrin Repeat and Zinc Finger Domain-Containing Protein 1, UniProt ID: ANKZ1_RAT
BTO: Brenda Tissue Ontology
C2h1orf52: Similar to Human Chromosome 1 Open Reading Frame 52 Protein, UniProt ID: D4A6E8_RAT
C5h8orf34: RIKEN cDNA A830018L16 Gene Protein, UniProt ID: D3ZPH6_RAT
Cadm1: Cell Adhesion Molecule 1, UniProt ID: CADM1_RAT
CRF: Continuous reinforcement schedule
Dguok: Deoxyguanosine Kinase, UniProt ID: A0A0G2K478_RAT
DIA: Data-independent acquisition
EBC: Effort-based choice
FDR: False discovery rate
GEE: Generalized estimating equations
GH: Growth hormone
Gnai1: Guanine Nucleotide-Binding Protein G(i) Subunit Alpha-1, UniProt ID: GNAI1_RA
Gnai3: Guanine Nucleotide-Binding Protein G(i) Subunit Alpha-3, UniProt ID: GNAI3_RAT
GO: Gene Ontology
GPCRs: G protein-coupled receptors
Ina: Internexin Neuronal Intermediate Filament Protein Alpha, UniProt ID: G3V8Q2_RAT
Irs2: Insulin Receptor Substrate 2, UniProt ID: F1MAL5_RAT
KEGG: Kyoto Encyclopedia of Genes and Genomes
LDN: Low-dose naltrexone
Map2k6: Mitogen-Activated Protein Kinase Kinase 6, UniProt ID: Q925D6_RAT
Mapk8: Mitogen-Activated Protein Kinase 8, UniProt ID: MK08_RAT
Mdga2: MAM Domain-Containing Glycosylphosphatidylinositol Anchor Protein 2, UniProt ID: MDGA2_RAT
Mycbp2: RCR-Type E3 Ubiquitin Transferase, UniProt ID: D4A2D3_RAT
PANTHER: Protein Analysis THrough Evolutionary Relationships
PASEF: Parallel accumulation serial fragmentation
POMC: Proopiomelanocortin
PR: Progressive ratio schedule of reinforcement
Rpl23: 60S Ribosomal Protein L23, UniProt ID: RL23_RAT
Stat5b: Signal Transducer and Activator of Transcription 5B, UniProt ID: STA5B_RAT
STRING: Search Tool for the Retrieval of Interacting Genes/Proteins
Sytl5: Synaptotagmin-Like Protein 5, UniProt ID: SYTL5_RAT
TCEP: Tris(2-carboxyethyl)phosphine
TIMS: Trapped ion mobility spectrometry
Tomm22: Mitochondrial Import Receptor Subunit TOM22 Homolog, UniProt ID: TOM22_RAT
UniProt: Universal Protein Resource
VTA: Ventral tegmental area
Zfp385a: Zinc Finger Protein 385A, UniProt ID: A0A0G2KB91_RAT
