# Supplementary material for: Naltrexone dose-selectively modulates goal-directed behavior and the hypothalamic proteome in rats
Source: Pharmacol Rep. 2025 May 28;77(4):983–98. doi: 10.1007/s43440-025-00735-4 (PMC12241218; doi:10.1007/s43440-025-00735-4)

1. **BEHAVIORAL EXAMINATION**

The data supporting the findings of this study are available in the Mendeley Repository at DOI: 10.17632/dxrfgrz9hf.1. Further information is available from the corresponding author upon request.

- 1. **Choice of outliers**


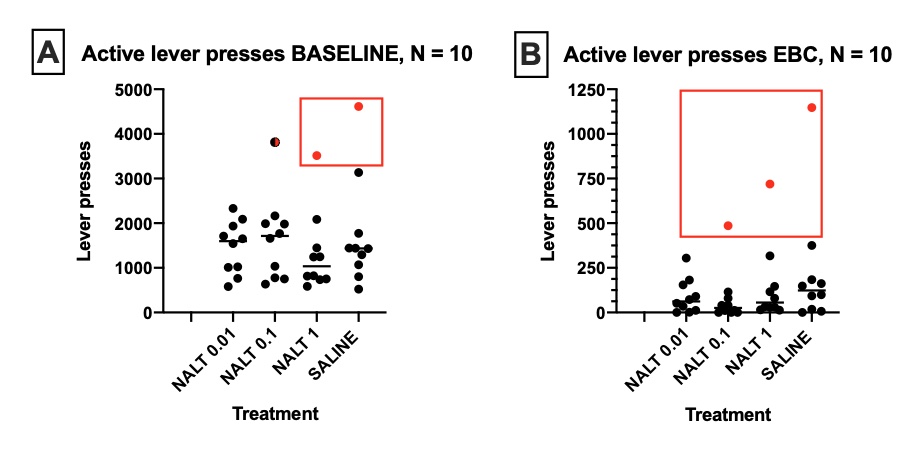
The Iterative Grubbs (Alpha = 0.01) test for outliers indicated three rats (see Figure 1A) based on the number of lever presses under EBC (deviation from mean > 2.5 standard deviations; one from each group: saline [ID = 38], naltrexone 0.1 [ID = 33], and 1 mg/kg [ID = 35]). Rats 35 and 38 were also indicated as outliers based on the baseline number of PROG lever presses (see Figure 1B).

Figure 1 Outliers indicated by Iterative Grubbs (Alpha = 0.01) test for outliers: (A) based on baseline PR response and (B) based on test EBC responses.

- 1. **Raw data and analysis**

GEE is best suited for longitudinal studies, enables different than normal data distribution and can be used to analyze correlated data. The statistical significance (P < 0.05) of factors was inferred based on estimated model effect, margins and marginal effects (average outcome variable level in a given group at a given time point) with respect to reference categories (vehicle treatment and first training session).

Here, **active lever presses** presented right-skewed distribution, as rats were more likely to achieve lower breakpoints both in PROG and EBC. In EBC, even some rats chose free food over a palatable reward that manifested as a press number even equal to zero. Because of abundant but relevant 0 values, we considered lever presses as an ordinal variable and employed a model of negative binomial distribution with a log link function. The statistical significance of factors was inferred based on estimated model effect, margins and marginal effects (average outcome variable level in a given group at a given time point) with respect to reference categories (vehicle treatment and first training session).

**Chow consumption** was correlated with session length, which depended on lever presses (the session was terminated if a rat did not press the lever for 10 consecutive min). Naturally, the longer the session was, rats the chow intake increased. To control the effect of time upon chow consumption, we normalized this parameter and used chow intake per session minutes instead [g/min], for detail see Figure 2. Data were ln transformed and then tested with the GEE linear model for continuous variables (session x treatment) of a normal distribution with an identity link function. The statistical significance of factors was inferred based on estimated margins and marginal effects with respect to reference categories (saline treatment and first EBC session).


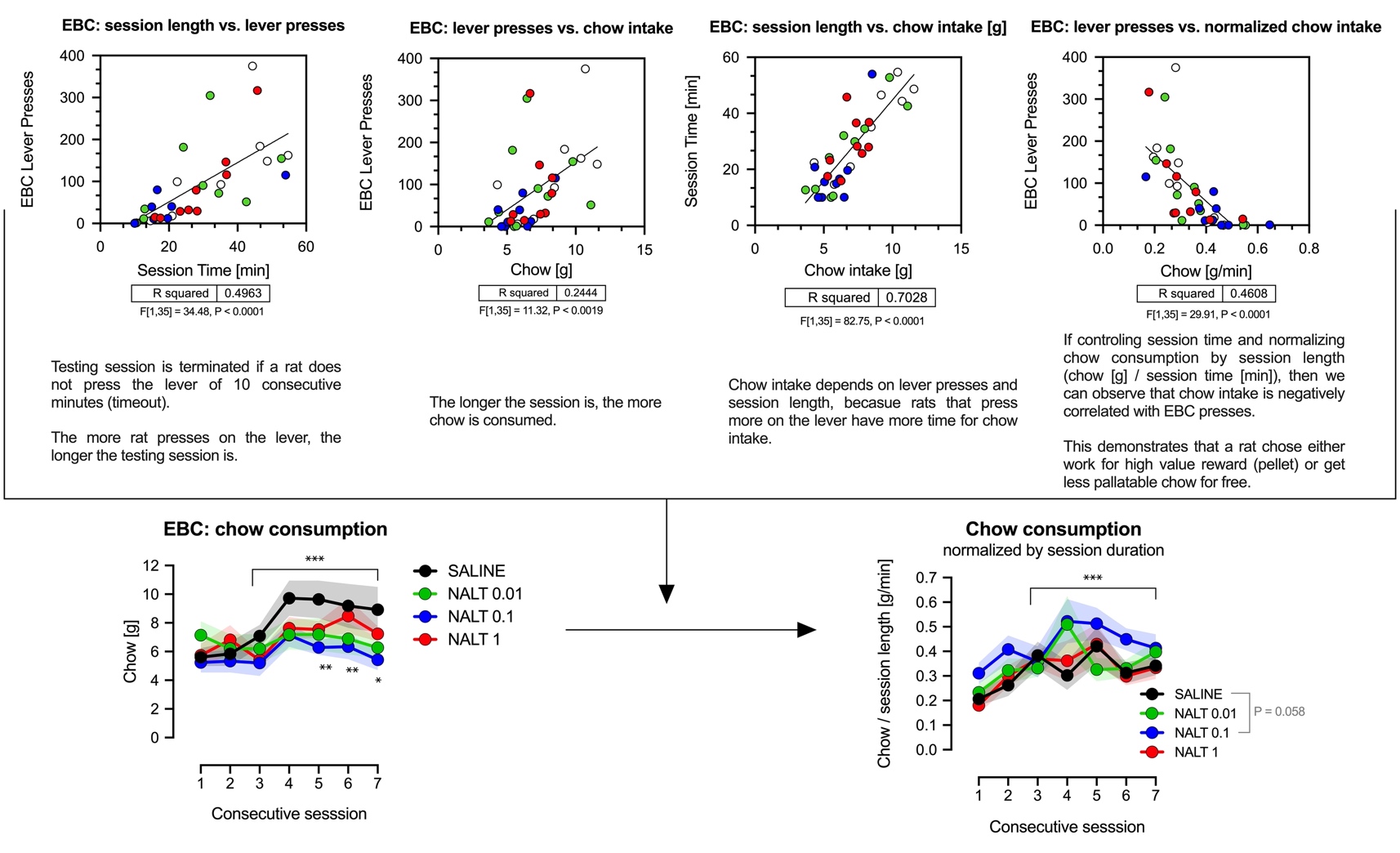


Figure 2 Normalization of free chow consumption step-by-step. Graphs’ description is available in the figure.

Statistical analysis for ‘EBC: chow consumption: chow [g] was done accordingly to method used for normalized chow consumption, see the text above the figure. Statistics is available in source files provided in the text. P < 0.05 was considered statistically significant.

Raw data are collected in *‘Raw data.xlsx’.* This file also contains SPSS output. Analysis was done using SPSS syntax *‘GEE template syntax.sps*’.

Original datafiles for SPSS analyses are available as *‘GEE ACTIVE LEVER PRESSES.sav’ and ‘GEE CHOW CONSUMPTION.sav’*. The original SPSS output is available in ‘GEE OUTPUT ACTIVE LEVER PRESSES.spv’ and ‘GEE OUTPUT CHOW CONSUMPTION.spv’, as well as PDF files.


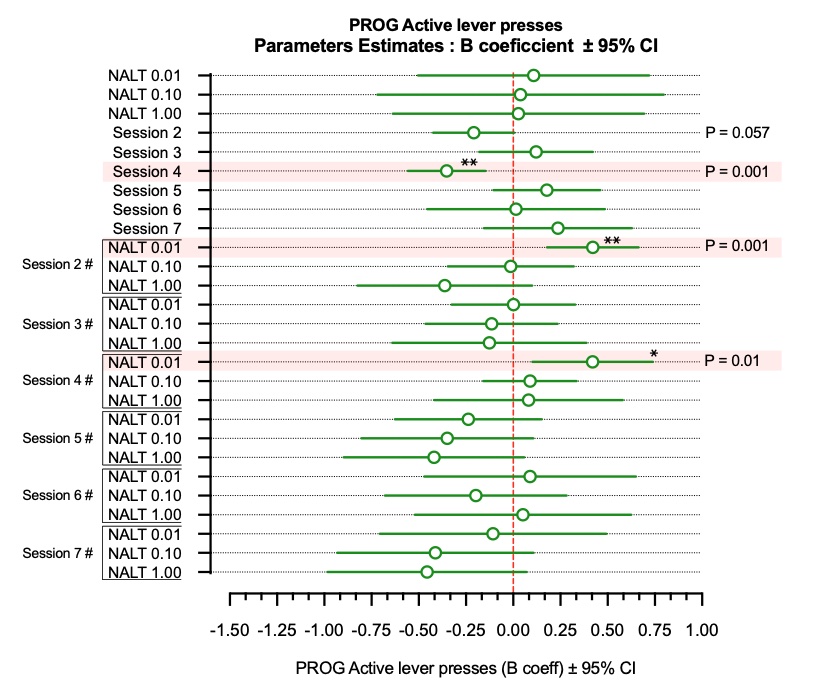

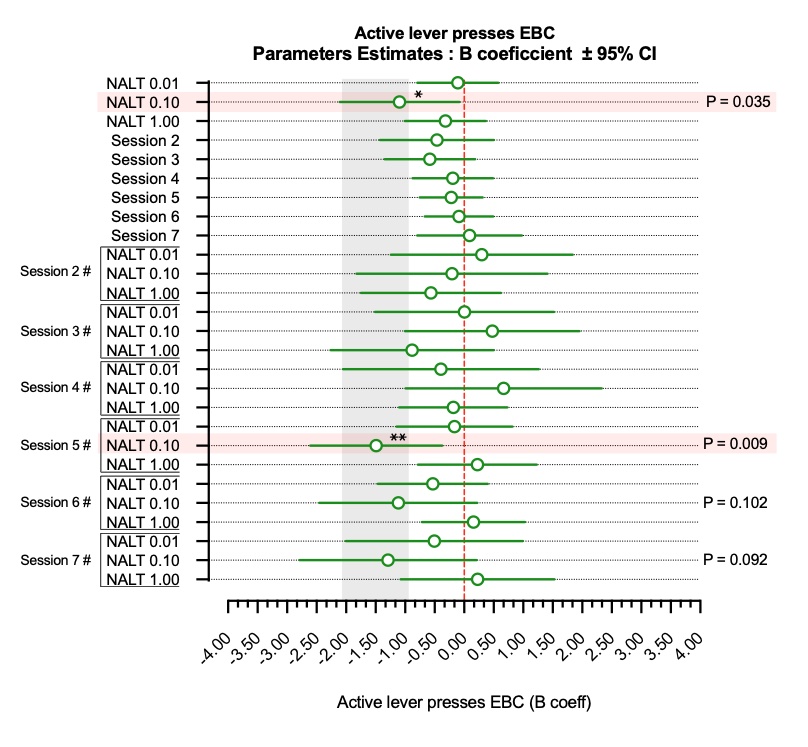
Beta coefficients and their 95%CI are presented below. Significant versus vehicle, session 1 or vehicle x session 1.


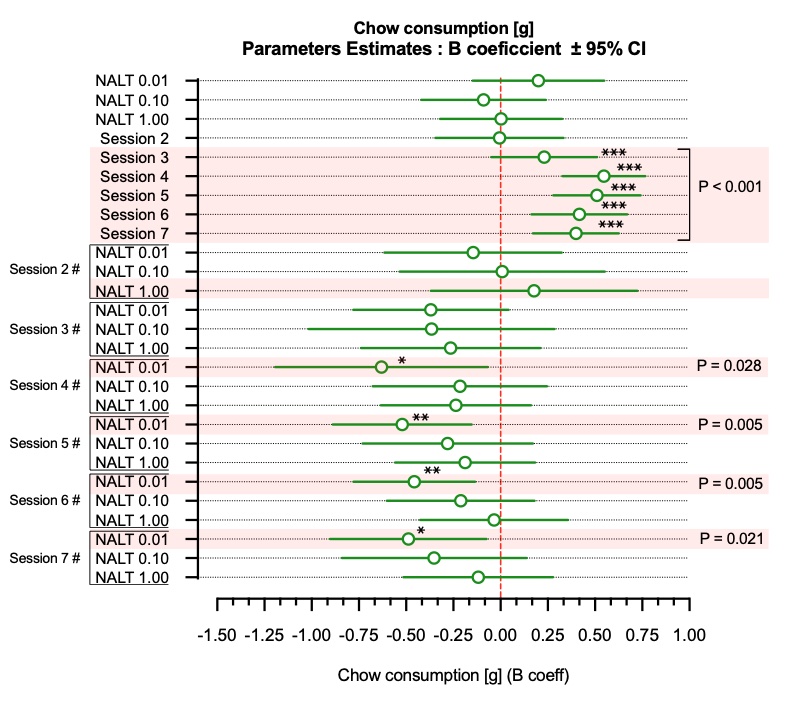

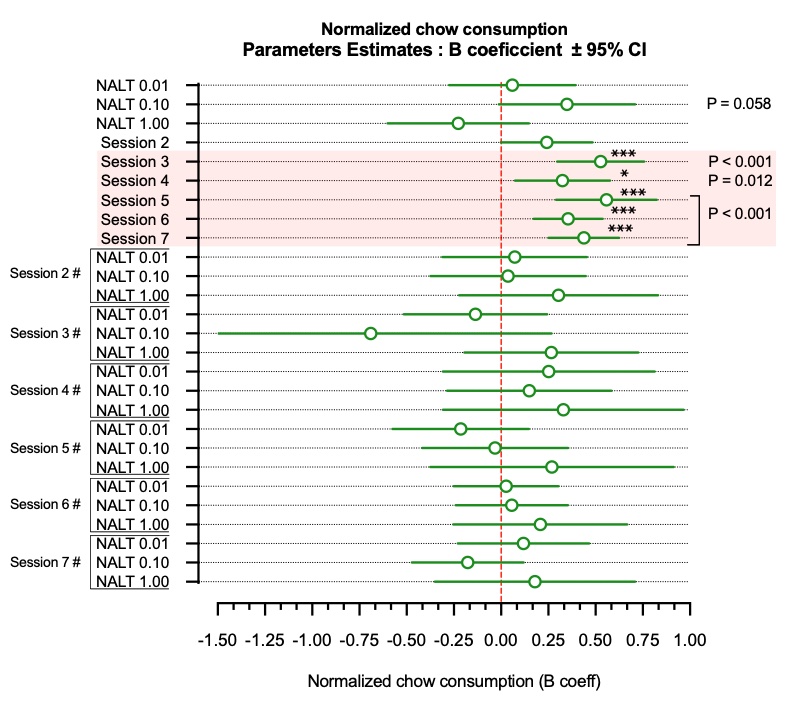


**Premises for using the GEE model**

1.
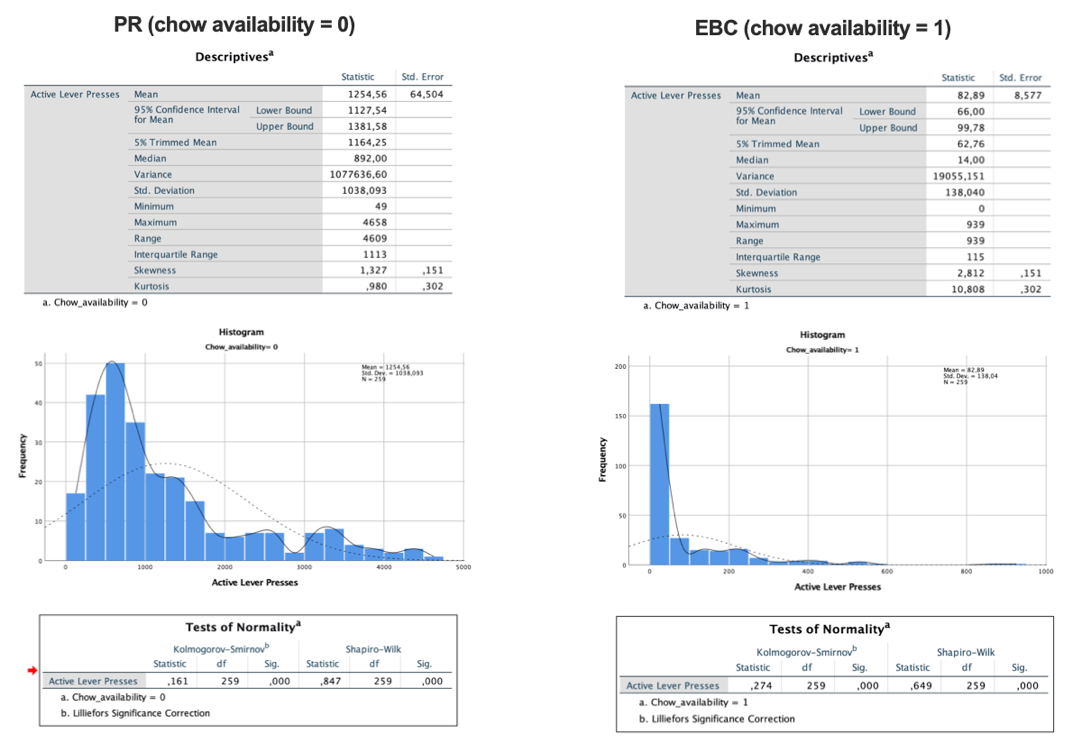
The data (br`eakpoint, number of lever presses, and pellets gained) were significantly right-skewed, meaning that rats were more likely to perform fewer lever presses, earn fewer pellets, and reach lower breakpoints in both PROG and EBC. This violated the assumption of normal data distribution and resulted in high standard deviations. In such cases, ANOVA may produce misleading conclusions rather than accurately assessing the effects of the tested factors. Below is presented the skewed distribution of active lever presses collected across all PROG (PR) and EBC test sessions. Given that the mean number of lever presses in PROG was 15 times higher than in EBC, we also opted for separate analyses to avoid underestimating the magnitude of the treatment effect in EBC.
2. To address the issue (1), we attempted to transform the data to achieve a normal distribution. Despite applying several transformations, the data still deviated significantly from the Gaussian standard (see the results of the Ln transformation below for reference).


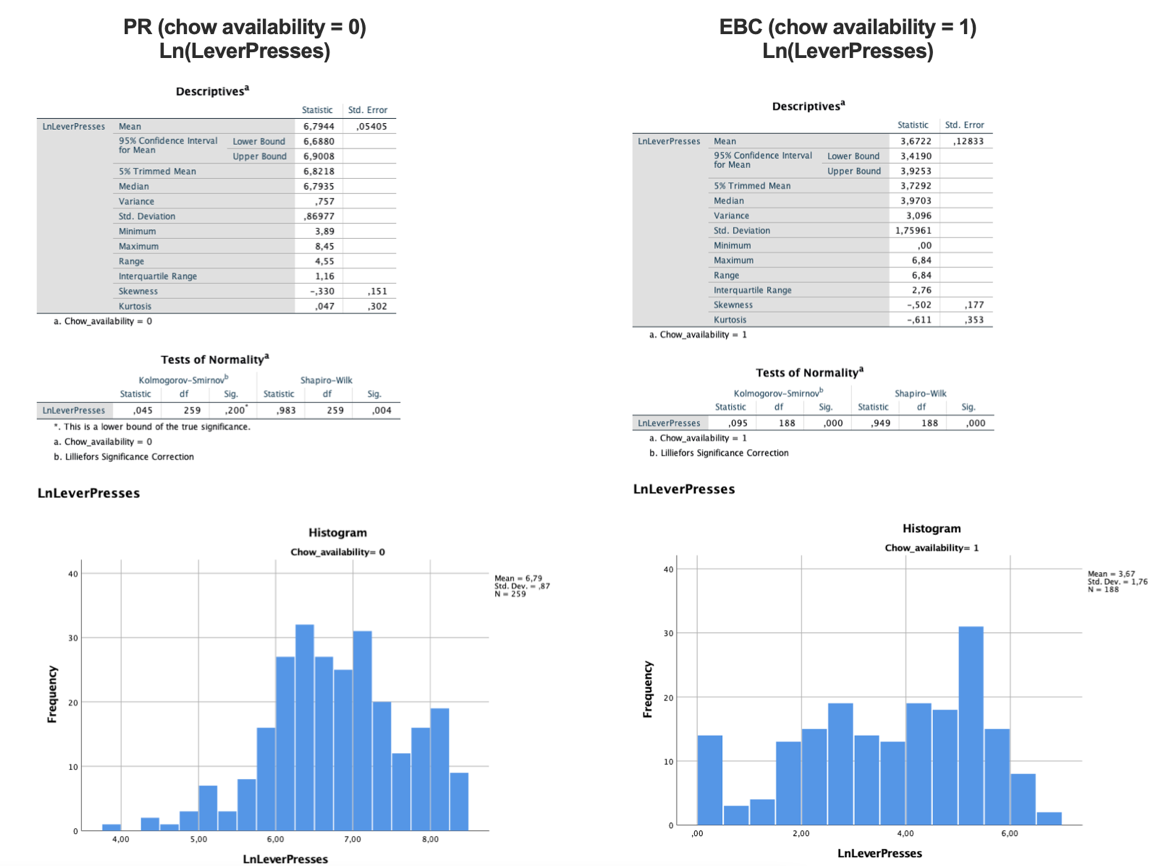


1. Transformations posed another challenge—most rely on quotients, logarithms, or trigonometric functions, which cannot appropriately handle '0' values. In EBC, '0' lever presses convey an important meaning, indicating that a rat prefers free food over a palatable pellet and perceives the effort as unprofitable. Therefore, it was essential to include '0s' in the analysis. However, in ANOVA on transformed data, these '0s' became irrelevant, leading to biased results.
2. Last but not least, we wanted to retain the repeated measures factor to observe how the effects of naltrexone evolved over time. Instead of performing multiple non-parametric equivalents of one-way ANOVA, we opted for an approach that could assess treatment effects over a longer measurement period, which here manifested in skewed and overdispersed datasets. This type of analysis is facilitated by the generalized estimating equations (GEE) model. GEE allows for the *a priori* definition of data distribution and properly accounts for repeated or correlated data. Since lever presses are count data, and we observed a skewed and overdispersed distribution (variance >> mean), we assumed a negative binomial (Pascal) distribution model. We then compared the goodness of fit across several models (some of them shown in the image below) and found that the chosen model provided the best fit.

**All these made ANOVA inapplicable for our dataset**.


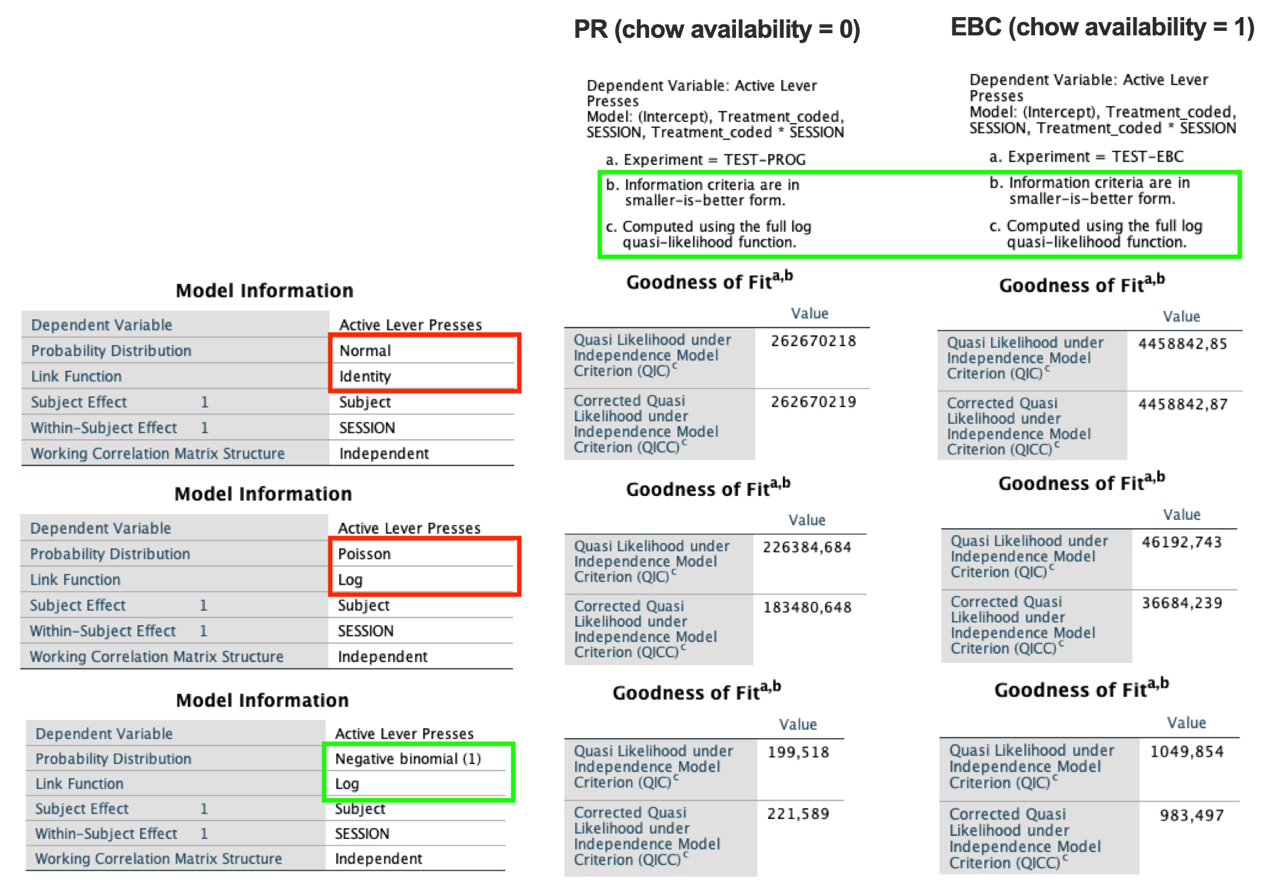

Supplement: Supplementary file 1 — Supplementary Material 1 [file 43440_2025_735_MOESM1_ESM.docx]
